# Changing the Landscape of the HIV Epidemic among MSM in China: Results from Three Consecutive Respondent-Driven Sampling Surveys from 2009 to 2011

**DOI:** 10.1155/2014/563517

**Published:** 2014-01-20

**Authors:** Xuefeng Li, Hongyan Lu, Catherine Cox, Yuejuan Zhao, Dongyan Xia, Yanming Sun, Xiong He, Yan Xiao, Yuhua Ruan, Yujiang Jia, Yiming Shao

**Affiliations:** ^1^State Key Laboratory for Infectious Disease Prevention and Control, National Center for AIDS/STD Control and Prevention, Chinese Center for Disease Control and Prevention, Collaborative Innovation Center for Diagnosis and Treatment of Infectious Diseases, No. 155 Changbai Road, Changping District, Beijing 102206, China; ^2^Karamay Center for Disease Control and Prevention, Karamay 834000, China; ^3^Beijing Center for Disease Control and Prevention, Beijing 100013, China; ^4^School of Public Health, University of Maryland, College Park, MD 20742, USA; ^5^Department of Preventive Medicine, Vanderbilt University School of Medicine, Nashville, TN 37232, USA

## Abstract

This study assessed the changes of HIV incidence and its predictors among Beijing's men who have sex with men (MSM). Three consecutive cross-sectional surveys were carried out using a consistent respondent-driven sampling (RDS) approach in 2009, 2010, and 2011, respectively. Structured-questionnaire based interviews were completed with computer-assisted self-administration. Incident infection was examined with BED capture enzyme immunoassay (BED-CEIA). The overall rate of HIV prevalence was 8.0% in the three years (95% confidence interval [CI]: 4.9%–11.2%). The overall rate of BED-CEIA incidence was 7.8/100 person years (PY) (95% CI: 5.5–10.1) with 6.8/100PY (95% CI: 3.4–10.2) in 2009, 11.2/100PY (95% CI: 6.2–16.3) in 2010, and 5.8/100PY (95% CI: 2.4–9.3) in 2011, respectively. Multivariable logistic regression analysis revealed that, compared with HIV-negative MSM, recently infected MSM were more likely to be bisexual (adjusted odds ratio [AOR] = 2.1, 95% CI: 1.1–4.1), live in Beijing ≤3 years (AOR = 2.1, 95% CI: 1.2–4.0), and have a negative attitude towards safe sex (AOR = 1.1 per scale point, 95% CI: 1.0–1.1). This study demonstrated a disturbing rise of HIV infections among Beijing's MSM. These findings underscored the urgency of scaling up effective and better-targeted intervention services to stop the rapid spread of the virus.

## 1. Introduction

Historically, the HIV epidemic in China has been confined to certain high-risk populations such as injection drug users (IDUs) and former blood and plasma donors in geographically disparate rural areas [1–6]. In recent years, the synthesized data suggested that HIV transmission has begun to shift from IDUs to populations at risk through unprotected sex, either through heterosexual contacts or male homosexual sex, accounting for nearly half of all new infections in 2007 [7]. Prevalence of HIV and other sexually transmitted diseases (STDs), for example, syphilis, is rapidly increasing among MSM in major cities [8–17]. MSM have emerged as a high-risk group for HIV in China. A meta-analysis revealed that the overall HIV prevalence among MSM in China increased substantially from 1.4% during 2001–2003 to 2.3% during 2004–2006 and to 5.3% during 2007–2009 [18]. The sentinel surveillance demonstrated that the overall HIV prevalence among MSM increased from 0.9% in 2003 to 6.3% in 2011 [19].

HIV prevalence is one of the key indicators to describe the disease burden. However, using the prevalence alone is difficult to capture the dynamic changes of the HIV epidemic, especially in the context of the expansion of antiretroviral treatment along with a spectrum of preventive intervention activities combined with a variety of behavioral, social, and structural factors that affected the spread of the virus. The emerging epidemic among MSM underscores the urgent need for the incidence measurement. Monitoring HIV incidence would pinpoint current dynamics of HIV infection and optimize the resource allocation for prevention activities that would result in the most HIV infections averted, resulting in the lowest HIV transmission rate possible.

There are three main ways to calculate HIV incidence. The “gold standard” method is a prospective cohort study; however, its disadvantages are it is expensive, difficult to maintain retention rate, lack of representative sampling, and the Hawthorne effect which affects a subject's behavior and may produce a bias conclusion [20, 21]. The second method is the mathematical model based on HIV prevalence data, but that relies on locally appropriate mortality data and survival time distribution [22]. Comparatively, laboratory-based methods for incidence estimation in a single cross-sectional survey have more reliability and attraction [20]. One commercialized and widely used assay to detect and distinguish recent from long-term infections is BED capture enzyme immunoassay (BED-CEIA), which has been applied to different populations worldwide [23–26]. In this study, we assessed the rates of recent HIV infections and its correlates using the BED-CEIA among MSM.

## 2. Methods

### 2.1. Study Design, Recruitment, and Instrument

Three consecutive cross-sectional RDS surveys were conducted among Beijing MSM from September to October 2009, from October 2010 to early January 2011, and from September to December 2011, respectively. The criteria for recruiting participants included men who had a valid recruitment coupon or who were selected as a seed, had engaged in homosexual behavior in the past 12 months (sex was defined as oral, anal, or mutual masturbation), currently living or working in Beijing, were at least 18 years old, and were able to provide written, informed consent. Structured questionnaire based interviews were conducted in an HIV voluntary counseling and testing clinic (VCT) located at the Beijing Center for Disease Control and Prevention.

Respondent-driven sampling (RDS) was used to recruit participants for each cycle, of which the details of the sampling method have been previously described [27]. Briefly, each recruitment chain started with 7–10 selected “seeds” that were given three coupons to recruit up to 3 eligible peers from their social network, who in turn were screened, enrolled, interviewed, were asked to refer 3 participants, and so on until both sample size and equilibrium were reached. Seeds were chosen from diverse networks with respect to geography (e.g., bar, bathhouse, park, and internet), demographic characteristics, and subculture, and they should be sociometric stars, articulate, and motivated. There are 7, 10, and 8 seeds in 2009, 2010, and 2011. Repeated seeds were 3, 3, and 2 between 2009 and 2010, between 2009 and 2011, and between 2010 and 2011, respectively. After signing the informed consent, we used computer-assisted self-administration to collect information on demographic characteristics, HIV risk behaviors, HIV testing history, drug use, access to HIV-related prevention services, and attitudes and perceptions of abilities for safe sex, where the psychosocial scale has been described previously [28]. This was assessed by a 15-item scale asking participants' self-efficacy in having safe sex (strongly disagree; somewhat disagree; somewhat agree; strongly agree), where the increasing scale score means greater negative attitudes and perceptions of abilities towards safe sex, and the internal reliability was high with a Cronbach's alpha value of 0.94 in our study. Being a bisexual was defined as someone who was married and/or self-reported to ever have had sex with a woman.

At the completion of the interview, a blood sample was obtained to test for HIV and syphilis. Participants were given 30 RMB for completion of the survey and 20 RMB for recruitment of their peers as monetary incentive. Pre- and postcounseling were offered to all participants and testing results were given within 1 to 3 weeks. At the time of specimen collection, the participant was given an appointment card with the name, telephone number, and email of the counselor, as well as the date, time, and location of their appointment for results counseling. Alternatively, the participant can arrange to contact the trained research staff for their results by telephone. BED results were not provided for participants because the main function of BED assay is to estimate population incidence rather than diagnose recent infection for an individual [20]. Those who were tested positive for HIV or syphilis were referred to appropriate medical and mental health and social support services as needed.

### 2.2. Laboratory Methods

Blood samples were tested for HIV and syphilis antibodies. HIV enzyme immunosorbent assay (Vironostika HIV Uni-Form plus O, bioMerieux, The Nether-lands) was used for HIV screening with a Western blot for confirmation (HIV Blot 2.2 WBTM, Genelabs Diagnostics, Singapore). Syphilis infection was determined using a rapid plasma reagin (RPR) test (Shanghai Rongsheng, China) and confirmed by the Treponema pallidum particle assay (TPPA) test (Fujirebio Inc., Japan). The BED IgG-capture ELISA (BED-CEIA, Calypte Biomedical Corporation, Rockville, Maryland) was applied to HIV-positive specimens for the detection of recent HIV seroconversions and estimation of HIV incidence. The basis of the assay is that it measures the ratio of HIV-specific IgG to total IgG, where the proportion slowly rises following seroconversion providing an indicator of disease progression [29]. Samples that had a normalized optical density (OD) less than or equal to the cutoff of 0.8 on the BED assay were classified as recent infections (seroconversion occurring ≤153 days), and others were considered as long-term infections. The calculation of HIV incidence followed US CDC's recommended formulas.

### 2.3. Statistical Analyses

Statistical analysis was performed using SAS version 9.1, where results with a *P* < 0.05 were considered significant. Of note, MSM who participated in the survey repeatedly were excluded from analysis in order to estimate the trends of HIV infection accurately. Chi-squared test was applied to compare sample demographic and behavior characteristics across survey years. To explore risk factors associated with recent HIV infection, we compared sociodemographic characteristics and behaviors of recently infected MSM to those of HIV-negative MSM using univariate logistic regression and forward stepwise multivariate logistic regression. Variables that were statistically significant in univariate analysis (*P* < 0.05) were entered into multivariate analysis. Considering the current limitations of RDSAT for conducting regression analyses [30] and the fact that the weighted RDS was performed for three individual years and no different results were found compared with the unweighted analytical method, it was determined that the time gap would have an effect on the sampling population and therefore we did not use weighted RDS analysis for the combined three cycles of data.

The study protocol and informed consent forms were reviewed and approved by the Institutional Review Boards of the National Center for AIDS/STD Control and Prevention (NCAIDS) in China, Vanderbilt University, and the University of California, San Francisco.

## 3. Results

### 3.1. Characteristics of Participants

A total of 501, 501, and 502 subjects were recruited in 2009, 2010, and 2011, respectively. Of the 1504 MSM assessed and screened in the three consecutive surveys, 4 participants were excluded due to being younger than 18 years old (1 participant in 2009), reporting no sexual behavior in the past year (1 participant in 2010), and having no valid recruitment card (2 participants in 2011). A total of 1500 eligible participants completed the questionnaire and provided blood samples. In the second cycle, 115 MSM participated in the first cycle repeatedly, and, in the third cycle, 57 participated in the first cycle and 28 participated in the second cycle (12 MSM participated in both the first and second cycle). As noted previously, 188 repeated participants were removed from the final sample for analysis.

Demographic and behavioral characteristics of study participants for each cycle are presented in Table 1. Major variables are different across the 3 cycles without a clear trend. In the subjects, more college or higher education level, employed, and urban MSM were recruited across the survey years. The proportion of MSM who were aware of the HIV status of the most recent male partner increased over the years from 26.4% in 2009 to 37.5% in 2011, and the proportion reporting knowing where to get an HIV test also rose from 94.2% in 2009 to 100.0% in 2011. The proportion reporting having a female sexual partner in the past 6 months decreased from 20.8% in 2009 to 10.8% in 2011, whereas the rates of unprotected sex with male or female partners both remained stably high.

### 3.2. HIV/Syphilis Prevalence and BED Estimated Incidence

Among 1312 participants, it was determined that 104 were infected with HIV and 1208 were HIV-negative. According to BED results, of the 104 HIV positive MSM, 45 were categorized as recently infected and 59 were long-term infections. The ratio of incidence infection to overall infection for 3 survey years was 41.7% (15/36), 50% (19/38), and 36.7% (11/30), respectively. The overall rate of BED-CEIA incidence was 7.8/100 person years (PY) (95% confidence interval (CI): 5.5–10.1) with 6.8/100 PY (95% CI: 3.4–10.2) in 2009, 11.2/100 PY (95% CI: 6.2–16.3) in 2010, and 5.8/100 PY (95% CI: 2.4–9.3) in 2011, respectively. Overall, HIV prevalence rose from 7.2% in 2009 to 9.9% in 2010 and dropped to 7.0% again in 2011 yet remaining at a high level, while, at the same time, syphilis prevalence decreased from 22.0% in 2009 to 12.2% in 2010 and 10.5% in 2011.

### 3.3. Factors Associated with Recent HIV Infection

Table 2 presents the univariate and multivariate logistic analyses of variables associated with recent HIV infection among Beijing MSM. Multivariate logistic regression analyses indicated that compared with HIV-negative MSM, recently infected MSM were significantly more likely to be bisexual (adjusted odds ratio (AOR) = 2.1, 95% CI: 1.1–4.1), live in Beijing ≤3 years (AOR = 2.1, 95% CI: 1.2–4.0), and have a negative attitude towards safe sex (AOR = 1.1 per scale point, 95% CI: 1.0–1.1).

## 4. Discussion

This study revealed the alarmingly high HIV prevalence and incidence rates among Beijing MSM in the past three years. The prevalence is higher than the rates found by previous RDS surveys among Beijing MSM (0.4% in 2004, 4.6% in 2005, and 5.8% in 2006) [31]. The BED-CEIA estimated incidence is much higher than previous rates estimated in either cross-sectional studies in 2005-06 (2.9/PY in 2005, 3.6/PY in 2006) [32] or cohort study in 2007 (2.6/PY) in the same population in Beijing [16], but the rates found in this study are similar to those reported from several cities, such as 7.01%, 7.98%, and 7.8% for Chongqing from 2006 to 2008 [33], 15.43% in 2009 [34], and 7.54% for Jiangsu in 2008 [35]. MSM as a high-risk population in China are facing a severe challenge against HIV infection, and the synthesized data revealed a disturbing expansion in the epidemic among MSM in China. The findings from these three consecutive surveys signals the beginning in changing the landscape of the HIV epidemic in China. HIV transmission has begun to shift from the historically main transmission route, IDU, to populations at risk through unprotected heterosexual contacts or male homosexual sex. The unprotected sex between men has been emerging as a major route of HIV transmission in China like many other nations surrounding China which experienced a resurgence in HIV transmission among MSM [36, 37].

In this study, HIV prevalence and BED-CEIA incidence estimates were very close, and the ratio of incidence infection to overall infection for 3 survey years was between 40% and 50%, which might demonstrate that the recent infection contributes to the main cause of the expanding HIV epidemic; therefore, it is important to identify the characteristics of recently infected MSM.

This study found that recently infected MSM were significantly more likely to be bisexual, and this finding is consistent with the conclusion from a recent meta-analysis, which indicated that bisexual behavior was significantly associated with a 30% increased risk of HIV infection [38]. In the present study, the three-year overall percentage of bisexual MSM and currently married MSM was 17.1% and 17.4%, respectively. The marriage rate was in agreement with the result from a systematic review which reported a national average of 17.0%, higher than most Western developed countries [39]. Chinese MSM suffer from both HIV-related and homosexuality-related stigma [40, 41], where they get married to cover their homosexual orientation, which when combined, these married MSM can be difficult to be reached and intervened. Common bisexual behaviors combined with inconsistent condom use put themselves and their female partner at high risk for HIV exposure. Further research is needed to understand the bridging effect of transmitting HIV from MSM to their female partners.

This study also found that MSM living in Beijing ≤3 years compose a risk group of recent HIV infection. Compared with longtime residents, recent migrants may lack HIV-related knowledge, have a low level of HIV infection awareness, and lack information about HIV/AIDS-related healthcare services, such as voluntary counseling and testing, condom distribution programs, and free antiretroviral therapy. Recent migrants are at particular risk for HIV infection; therefore, it is important to improve their access to HIV prevention and treatment services, and further study is needed to explore the differences between longtime and short-term residents.

Previously published studies among Chinese or foreign MSM indicated that unprotected anal sex was a predictor of recent HIV infection [42, 43], and, although we did not find that relationship in our study, we found that holding a negative attitude towards safe sex practices was associated with recent HIV infection. In the three annual cross-sectional surveys, more than two-thirds of MSM had multiple male sex partners in the past 6 months, and about two-fifths reported unprotected insertive or receptive anal sex in the past 6 months over the period. Given the common multiple sexual partnerships and low rate of condom use in this population, more health education and harm reduction programs are urgently needed to change their low perception of HIV infection risk and increase their confidence in practicing safer sex.

We recognized the limitations of the study. First, the questionnaire involves private and sensitive HIV risk behaviors, so participants may choose to give socially desirable answers instead of true effects, which might introduce information bias. Second, the cross-sectional study design cannot establish the causality between associated factors and recent HIV infection. Third, due to misclassification of long-term HIV infection as recent infection among persons who are on antiretroviral therapy or who have AIDS but with declining antibody levels, the BED assay may overestimate incidence rates [20, 44]. Lastly, although RDS is effective in reaching hard-to-reach populations, it is based on a dual incentive regime; therefore, financial reward may attract more low-income people to participate in the study which may limit the representativeness of the sample [30]. Although there are limitations in our study, it adds to our understanding of the dynamics of HIV infections among Beijing MSM.

This study, together with our previous study findings, demonstrated a disturbing rise of HIV infections among Beijing's MSM. HIV disproportionately affected MSM who were bisexual, short-term Beijing residents, with a negative attitude towards safe sex. These findings underscored the urgency of increasing effective and better-targeted intervention services to stop the rapid spread of the virus, which includes risk reduction education, condom promotion and distribution, encouraging early and frequent HIV testing, and expanding the coverage of HIV prevention measures. These interventions will help curb the spread of HIV infection inside and outside the MSM population.

## Figures and Tables

**Table 1:** Demographic and behavior characteristics of MSM, Beijing, China, 2009–2011.

Variable	2009 (*n* = 500)		2010 (*n* = 385)		2011 (*n* = 427)		*P*
	*N*	%	*N*	%	*N*	%	
Age group in years							
18–25	173	34.6	154	40.0	147	34.4	0.5934
26–35	205	41.0	156	40.5	186	43.6	
36–71	122	24.4	75	19.5	94	22.0	
Education, completed college or more	174	34.8	172	44.7	208	48.7	<0.0001
Married to a woman currently	104	20.8	56	14.6	68	15.9	0.0325
Living with boyfriend	104	20.8	74	19.2	101	23.7	0.2896
Employment status							
Employed	454	90.8	365	94.8	416	97.4	<0.0001
Unemployed	46	9.2	20	5.2	11	2.6	
Residence							
Urban	396	79.2	343	89.1	389	91.5	<0.0001
Rural	104	20.8	42	10.9	36	8.5	
Living three years or fewer in Beijing	221	44.2	168	43.6	184	43.1	0.9440
Mean monthly income (RMB), P12M							
None	58	11.6	41	10.7	30	7.0	<0.0001
<1000	47	9.4	19	4.9	11	2.6	
1000–2999	271	54.2	173	44.9	144	33.7	
3000–4999	80	16.0	64	16.6	137	32.1	
≥5000	44	8.8	88	22.9	105	24.6	
Have health insurance	236	47.2	209	54.3	229	53.6	0.0590
Self-reported sexual orientation							
Homosexual	346	69.2	278	72.2	313	73.3	0.0693
Heterosexual	5	1.0	7	1.8	2	0.5	
Bisexual	147	29.4	93	24.2	109	25.5	
Do not know	2	0.4	7	1.8	3	0.7	
Self-reported preferred role in anal sex with men							
Equally	291	59.4	235	63.3	234	56.0	0.3509
Insertive	133	27.1	91	24.5	124	29.7	
Receptive	66	13.5	45	12.1	60	14.4	
Had male sexual partner, P6M	488	97.6	373	96.9	427	100.0	0.0020
More than five male sex partners, P6M	144	28.8	55	14.3	73	17.1	<0.0001
Type of most recent male partner, P6M							
Regular	309	63.3	205	55.0	267	62.5	0.0281
Casual	179	36.7	168	45.0	160	37.5	
Venues of founding/meeting last male partner, P6M							
Internet/bar/massage/club	306	63.1	274	73.5	300	70.3	0.0033
Bathhouse/sauna/park/public rest room	179	36.9	99	26.5	127	29.7	
Venues of sex with last male partner, P6M							
Residence/bar/club/hotel	428	87.7	341	91.4	370	86.7	0.0894
Bathhouse/sauna/park/public rest room	60	12.3	32	8.6	57	13.4	
Unprotected insertive anal sex with last male partner, P6M	127	36.6	93	38.9	112	40.4	0.6118
Unprotected receptive anal sex with last male partner, P6M	115	40.4	99	50.0	95	42.8	0.1026
Knew the HIV status of most recent male partner	129	26.4	133	35.7	160	37.5	0.0007
Disclosed HIV status to most recent male partner	236	48.4	180	48.3	230	53.9	0.1727
Bisexual	104	20.8	55	14.3	66	15.5	0.0206
Ever had sex with a woman	283	56.6	181	47.0	207	48.5	0.0075
Had female sexual partner, P6M	104	20.8	49	12.7	46	10.8	<0.0001
Unprotected sex with most recent female partner, P6M	75	72.1	41	85.4	35	76.1	0.2024
Knows where to get HIV test	470	94.2	383	99.5	427	100.0	<0.0001
HIV testing history							
Never tested	166	33.2	148	38.4	93	21.8	<0.0001
Tested more than one year prior to interview	68	13.6	48	12.5	49	11.5	
Tested within the past year	55	11.0	55	14.3	49	11.5	
Tested within the past 6 months	142	28.4	102	26.5	197	46.1	
Tested within the past 3 months	69	13.8	32	8.3	39	9.1	
Received free condoms/lubricant, P12M	436	87.2	267	69.4	330	77.3	<0.0001
Bought condoms, P12M	249	49.8	234	60.8	246	57.6	0.0029
Drug use, P12M	4	0.8	2	0.5	4	0.9	0.7847
Smoking, P12M	217	43.4	154	40.0	153	35.8	0.0640
Level of HIV risk via homosexual contacts for self-perception							
Great	63	12.6	28	7.3	42	9.8	0.4414
Moderate	92	18.4	80	20.8	75	17.6	
Small	286	57.2	202	52.6	272	63.7	
None	59	11.8	74	19.3	38	8.9	
Current test HIV positive	36	7.2	38	9.9	30	7.0	0.2433
Current test syphilis positive	110	22.0	47	12.2	45	10.5	<0.0001

*RMB: renminbi, approximately US $0.15; P12M: in the past 12 months; P6M: in the past 6 months; chi-squared test was used to compare proportions.

**Table 2:** Risk factors associated with recent HIV infection among MSM, 2009–2011 (recent HIV infection versus HIV negative, *N* = 1253).

Variable	Recent infection % (*N*)	OR (95% CI)	*P*	AOR (95% CI)	*P*
Age group in years					
18–25	3.5 (16)	1.0			
26–35	3.3 (17)	0.9 (0.5–1.9)	0.8227		
36–71	4.4 (12)	1.2 (0.6–2.7)	0.5706		
Education					
College or above	3.1 (17)	1.0			
High school or less	3.9 (28)	1.3 (0.7–2.3)	0.4575		
Current marital status					
Unmarried	3.1 (32)	1.0			
Married	6.0 (13)	2.0 (1.0–3.9)	0.0386		
Living with boyfriend					
Yes	1.5 (4)	1.0			
No	4.2 (41)	2.9 (1.0–8.1)	0.0453		
Employment status					
Employed	3.7 (44)	1.0			
Unemployed	1.5 (1)	0.4 (0.1–2.9)	0.3600		
Residence					
Urban	3.6 (39)	1.0			
Rural	3.5 (6)	1.0 (0.4–2.3)	0.9219		
Years living in Beijing					
>3	2.5 (18)	1.0		1.0	
≤3	5.0 (27)	2.0 (1.1–3.7)	0.0221	2.1 (1.2–4.0)	0.0153
Mean monthly income (RMB), P12M					
≥1000	3.6 (38)	1.0			
<1000	3.7 (7)	1.0 (0.5–2.3)	0.9523		
Have health insurance					
Yes	3.2 (21)	1.0			
No	4.0 (24)	1.2 (0.7–2.3)	0.4771		
Self-reported sexual orientation					
Others	4.1 (15)	1.0			
Homosexual	3.4 (30)	0.8 (0.4–1.5)	0.5443		
Self-reported preferred role in anal sex with men					
Equally	4.6 (33)	1.0			
Insertive	2.1 (7)	0.4 (0.2–1.0)	0.0487		
Receptive	3.1 (5)	0.7 (0.3–1.7)	0.3875		
No. of male sex partners, P6M					
≤5	3.0 (30)	1.0			
>5	5.9 (15)	2.0 (1.1–3.8)	0.0318		
Type of most recent male partner, P6M					
Regular	3.3 (25)	1.0			
Casual	4.2 (20)	1.3 (0.7–2.3)	0.4473		
Venues of founding/meeting last male partner, P6M					
Internet/bar/massage/club	3.9 (33)	1.0			
Bathhouse/sauna/park/public rest room	3.2 (12)	0.8 (0.4–1.6)	0.5326		
Venues of sex with last male partner, P6M					
Residence/bar/club/hotel	3.3 (36)	1.0			
Bathhouse/sauna/park/public rest room	6.6 (9)	2.1 (1.0–4.4)	0.0553		
Unprotected insertive anal sex with last male partner, P6M					
No	2.7 (14)	1.0			
Yes	3.8 (12)	1.4 (0.6–3.1)	0.3894		
Unprotected receptive anal sex with last male partner, P6M					
No	4.8 (18)	1.0			
Yes	5.6 (16)	1.2 (0.6–2.3)	0.6490		
Knew the HIV status of most recent male partner					
Yes	3.1 (13)	1.0			
No	3.9 (32)	1.3 (0.7–2.4)	0.4914		
Disclosed HIV status to most recent male partner					
Yes	3.4 (21)	1.0			
No	4.0 (24)	1.2 (0.7–2.2)	0.5677		
Bisexual					
No	3.1 (32)	1.0		1.0	
Yes	6.1 (13)	2.0 (1.1–4.0)	0.0339	2.1 (1.1–4.1)	0.0317
Ever had sex with a woman					
Yes	4.1 (26)	1.0			
No	3.1 (19)	0.7 (0.4–1.4)	0.3445		
Had female sexual partner, P6M					
Yes	3.1 (6)	1.0			
No	3.7 (39)	1.2 (0.5–2.8)	0.7169		
Unprotected sex with most recent female partner, P6M					
No	4.4 (2)	1.0			
Yes	2.8 (4)	0.6 (0.1–3.5)	0.5990		
Received a test for HIV, P12M					
Yes	3.4 (24)	1.0			
No	3.9 (21)	1.2 (0.6–2.1)	0.5916		
Received free condoms/lubricant, P12M					
Yes	3.4 (33)	1.0			
No	4.5 (12)	1.4 (0.7–2.7)	0.3725		
Bought condoms, P12M					
Yes	4.4 (31)	1.0			
No	2.5 (14)	0.6 (0.3–1.1)	0.0768		
Received free STD examination or treatment					
Yes	3.8 (22)	1.0			
No	3.4 (23)	0.9 (0.5–1.7)	0.7632		
Received free VCT					
Yes	3.9 (25)	1.0			
No	3.2 (20)	0.8 (0.4–1.5)	0.4989		
Main resource of knowledge of HIV/AIDS					
Internet	3.4 (16)	1.0			
Friend/relative/sex partner/peer education/TV/newspapers/ doctors/VCT/school education	3.7 (29)	1.1 (0.6–2.0)	0.7657		
Smoking, P12M					
No	3.7 (28)	1.0			
Yes	3.5 (17)	0.9 (0.5–1.7)	0.8265		
Negative attitudes towards safe sex (Cronbach's alpha = 0.94, range = 15–60, median = 20)	na	1.1 (1.0-1.1)	0.0006	1.1 (1.0-1.1)	0.0012
Current test syphilis positive					
No	3.2 (34)	1.0			
Yes	6.0 (11)	1.9 (1.0–3.9)	0.0639		
Year					
2009	3.1 (15)	1.0			
2010	5.2 (19)	1.7 (0.8–3.4)	0.1351		
2011	2.7 (11)	0.9 (0.4–1.9)	0.7018		

*RMB: renminbi, approximately US $0.15; P12M: in the past 12 months; P6M: in the past 6 months; OR: odds ratio; CI: confidence interval; AOR: adjusted odds ratio; NA: not applicable.
